# Combined Impact of Nanoplastics and Temperature on Green Algae: Implications for Growth, Lipid Content and Organic Exudates

**DOI:** 10.1111/1758-2229.70246

**Published:** 2025-12-02

**Authors:** Sareh Yaripour, Sadikshya Ghimire, Alexey Ignatev, Raine Kortet, Rebecca Burkl, Jari T. T. Leskinen, Jussi V. K. Kukkonen, Jarkko Akkanen, Jukka Kekäläinen, Mohammad Salar Sohrabi, Ursula Strandberg

**Affiliations:** ^1^ Department of Environmental and Biological Sciences University of Eastern Finland Joensuu Finland; ^2^ Department of Biological and Environmental Science University of Jyväskylä Jyväskylä Finland; ^3^ Department of Technical Physics, SIB Labs University of Eastern Finland Kuopio Finland

**Keywords:** AOM production, fatty acids, green algae, growth, polymethylmethacrylate nanoparticles, warming

## Abstract

Freshwater ecosystems are under significant environmental stress due to warming and plastic pollution. However, our understanding of their combined effects on primary producers is scarce. We investigated the effects of plain spherical polymethylmethacrylate (PMMA) nanoparticles (NPs) and the acute temperature increases on the growth and fatty acid content of the freshwater green algae *Pseudokirchneriella* sp. over a 5‐day exposure period. The experiment was conducted at three NP concentrations (0.05, 0.5, and 5 mg/L) and two temperature levels (20°C and 25°C). We analysed algal organic matter (AOM) produced during the experiments. Higher temperature stimulated cell growth at lower NP concentrations, but not at the highest NP concentration. Fatty acid composition was affected by temperature but not by NPs. At 20°C, the fractions of low, intermediate, and high molecular weight (MW) AOM had a higher tryptophan‐like fluorescence, pointing to a higher protein‐like content. Humic‐like fluorescence of low MW AOM fractions was higher at higher temperature. At 25°C, the fluorescence response increased slightly with increasing NP concentrations. The findings suggest that temperature has a greater effect on altering fatty acid composition and AOM chemistry than NPs.

## Graphical abstract

The combined effects of nanoplastics and temperature on green algae were investigated. Higher NP concentration suppressed the temperature‐induced growth of the algae. Temperature plays a dominant role in influencing fatty acid composition and chemistry of AOM than exposure to NPs.

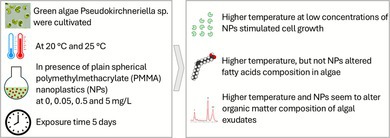

## 1 Introduction

Freshwater ecosystems are experiencing a range of environmental stressors, including, but not limited to, warming, eutrophication, and pollution, rendering them among the most stressed habitats worldwide (Woodward et al. 2010). Global warming and plastic pollution are among the main anthropogenic threats to aquatic ecosystems (Altshuler et al. 2011). Generally, these have been addressed as separate stressors, although plastic pollution is undoubtedly linked with climate change (Ford et al. 2022; Lins et al. 2022). Understanding the combined effects of multiple stressors is critical because co‐exposure may have additive, synergistic or antagonistic effects on biota (Hiltunen et al. 2021; Yang et al. 2021; Zhao et al. 2023). Global climate change is expected to lead to more frequent and intense heatwaves in surface waters (Woolway et al. 2021). These changes not only affect the metabolism of poikilothermic organisms but also alter water chemistry and hydrology (Adrian et al. 2009; Woodward et al. 2010; Mooij et al. 2005; Jeppesen et al. 2020; Woolway et al. 2021). Additionally, rising temperatures may increase the toxicity of environmental contaminants for organisms, as they can influence the accumulation efficiency of pollutants, like plastics, and pose challenges for detoxification and elimination processes (Lins et al. 2022). Plastics have been increasingly detected in diverse environments worldwide, including water, soil, sediment, and even the most remote areas of the Antarctic and Arctic (Materić et al. 2022). In the environment, plastics undergo degradation and fragmentation, forming smaller particles known as microplastics (MPs: < 5 mm) and nanoplastics (NPs: < 1000 nm) (Cai et al. 2021); MPs/NPs can also be released from numerous sources such as industrial processes and consumer cosmetics (Tallec et al. 2018; Shen et al. 2019). Nanoplastics have drawn intense attention recently due to their smaller size and greater ability to penetrate biological barriers, making them potentially more toxic than MPs (Shen et al. 2019). The estimated NP concentrations in the aquatic environments vary from 0.8 ng/L to 5 mg/L (Shi et al. 2024).

Phytoplankton are the primary producers at the base of the food chain, supporting upper trophic levels and maintaining the resilience and stability of aquatic ecosystems (Safi et al. 2014; Zhang et al. 2024). Stress‐induced changes in algal growth rate and biochemical quality, such as fatty acid (FA) composition, may decrease resource quantity and quality for upper trophic level consumers, such as zooplankton and fish (Guschina and Harwood 2006). Fatty acids are a significant source of energy and essential nutrients for primary consumers. Specifically, long‐chain polyunsaturated fatty acids (PUFAs) from the n‐3 and n‐6 families are almost solely produced by algae, and most animals have limited ability to synthesise these PUFAs *de novo*, but need to obtain them from the diet (Maltsev and Maltseva 2021). The n‐3 and n‐6 PUFAs, and their relative abundance, that is, n‐3/n‐6 ratio, affect survival, reproduction, and population growth of fish (Parrish 2009). Additionally, the imbalanced n‐3/n‐6 ratio in the western diet has been linked with cardiovascular diseases, cancer, and inflammatory and autoimmune diseases (Simopoulos 2002).

Algal organic matter (AOM) comprises various forms of polysaccharides, proteins, lipids, low molecular weight acids, and other dissolved organic substances (Bhaskar and Bhosle 2006; Villacorte et al. 2015). The quantity and quality of AOM produced by algae can vary in response to stressful conditions such as environmental contaminants, temperature fluctuations, and low nutrients (Maršálek and Rojíčková 1996). Additionally, bacteria or viruses invading the algae and the disruption and decay of algal cells can contribute to AOM production. Some algae may release these organic materials under normal conditions (Fogg 1983; Villacorte et al. 2015). AOM is a key component in nutrient cycling, microbial activity, water quality, and trophic interactions in the aquatic environment (Cole et al. 2007; Henderson et al. 2010; Li et al. 2019; Deng et al. 2023). Changes in the quality and quantity of AOM can substantially impact the health and function of aquatic ecosystems.

Our knowledge of the combined effects of the acute temperature increase and environmental pollutants, such as NPs, is limited. In this study, we examine the separate and combined effects of plain spherical polymethylmethacrylate (PMMA) NPs and the acute temperature increase on the growth and fatty acid composition of freshwater green algae *Pseudokirchneriella* sp. under laboratory conditions. Earlier studies have mainly focused on the effects of individual stressors, such as increased temperature or plastic exposure, on fatty acid profiles, but the knowledge of the possible combined effects of two major stressors on the biochemical composition of algae is currently very limited (Feijão et al. 2018; Sun et al. 2023). Additionally, we study AOM, produced in the experiments, using high‐performance size‐exclusion chromatography (HPSEC) with simultaneous UV and fluorescence detection, which is becoming one of the preferred choices for rapid characterisation of organic matter from natural environments (Derrien et al. 2019; Tuhkanen and Ignatev 2019). Since the exact composition of AOM and the magnitude of possible changes are unknown, a non‐target surrogate approach, such as HPSEC fractionation, may be helpful to detect NPs‐induced changes in the AOM chemistry. In this context, three hypotheses will be tested here. Firstly, we hypothesize that increased NPs exposure and warming will decrease the n‐3/n‐6 PUFA ratio. Secondly, we hypothesise that warming will alter the toxicity profile of PMMA NPs and decrease cell growth and fatty acid content. Thirdly, we hypothesise that exposure of algae to PMMA NPs and/or elevated temperature changes their metabolic rates, affects their growth rate, or induces other stress responses, and subsequently alters the composition of AOM.

## 2 Materials and Methods

### 2.1 The Model NPs

Plain (without additional coatings or modifications) polymethylmetacrylate (PMMA) NPs, 25 nm in nominal diameter, were purchased from Micromod Partikeltechnologie GmbH, Rostock, Germany. PMMA is among the most common types of plastic debris found in the environment. It is a form of strong acrylic plastic which is commonly used, for example, in plastic windows, smartphone screens, aquariums, medical devices, and fibre optic sensors (Ali et al. 2015). Despite its widespread use, little research has focused on its biological impact on aquatic organisms (Venâncio et al. 2019).

### 2.2 Particle Characterisation

The commercial PMMA particles were prepared using a negative staining protocol for ultra‐high‐resolution transmission electron microscopy. NP particles were mixed with distilled water to a final concentration of 0.5 mg/mL. The nanoplastics‐water solutions were sonicated for 300 s., and 10 μL of each NPs solution was dropped onto the standard copper grids (200 Mesh, 3.05 mm in diameter) with a formvar membrane. After 120 s. of incubation, the excess water was removed using filter paper. The grid sample was rinsed with five drops of distilled water and again dried using filter paper. 5 μL of 1.0% (w/w) aqueous uranyl acetate solution was applied to the specimens for 60 s. to obtain negative staining of the NPs. Finally, the excess solution was removed using filter paper, and the samples were dried at room temperature.

Nanoplastics were characterised using a 200 kV transmission electron microscope, TEM (Jeol JEM‐2100F, Jeol Ltd., Tokyo, Japan). They were micrographed up to 100,000 x magnification to estimate their particle size and study morphology using ImageJ 1.53 t software. The control sample was studied using TEM to characterise the imaging background of the empty TEM grid. In addition to TEM characterisation, the effective particle diameter of NPs was measured in 5.0 mg/mL of distilled water solution of NPs using DLS (Malvern Zetasizer Nano ZS) after vortex mixing and sonication for 60 s.

### 2.3 Algal Cultivation

Chlorophyte *Pseudokirchneriella* sp. (Selenastraceae) was used as a model organism to evaluate the combined effects of temperature and NPs exposure on the growth and fatty acid composition in algae, as well as the molecular size distribution of dissolved organic compounds exuded by the algae. *Pseudokirchneriella* (CHL‐57) was purchased from the Norwegian Culture Collection of Algae (NORCCA), which is hosted by the Research Institute for Water and the Environment (NIVA). Initially, the algae were grown in 800 mL Erlenmeyer flasks with Z8 medium according to the protocol of NORCCA (https://norcca.scrol.net/node/3963) in a temperature‐controlled chamber on a 16:8 h (L:D) cycle at 20°C. Flasks were randomly relocated every day and manually shaken every 6 h. Media preparation and all culture transfers were performed inside a laminar flow hood.

### 2.4 Experimental Design

In this full factorial experiment, we tested two stressors: the acute temperature increase, with two levels, 20°C and 25°C, and NPs, with four concentrations. The nanoplastic concentrations were: 0 mg/L (control), 0.05, 0.5, and 5 mg/L (representing relevant environmental concentrations, see Zhang et al. 2024). Each treatment contained five replicates. Before the exposure, the Z8 medium was prepared and kept at 20°C. In the experiment, algae were not allowed to acclimatise to the higher temperature, as the aim was to investigate the effects of the acute temperature stress. To prevent particle aggregation, stock dispersion containing 10 mg/mL of NPs was sonicated in a bath sonicator (35 kHz frequency, DT 255, Bandelin electronic, Sonorex digital, Berlin, Germany) for 10 min before starting the 5‐day exposure. The exposure of NPs and temperature increase consisted of two stages. In stage I, the corresponding media were exposed to the three concentrations of NPs for 24 h at 25°C. After exposure, each flask was stirred by a glass rod for 30 s to distribute particles equally. Then, flasks were covered with aluminum foil and placed in a temperature‐controlled chamber set at 25°C until NPs and media were equilibrated. Then, in stage II, we added algae cells adjusted at 4 × 10^5^ cells/mL to the exposed media in a final volume of 800 mL (per flask) and put the flasks back in the chamber for 5 days. The same procedure was followed, and flasks were kept in the chamber set at 20°C. The flasks were shaken manually three times a day and redistributed randomly every day.

### 2.5 Algal Growth

Cells were counted at days 1 and 5 from each replicate and used to calculate the specific growth rate (μ) as follows:

(1) $$\mu =\frac{\ln\left(N_{5}/N_{1}\right)}{t_{5}-t_{1}}$$

where *N*_5_ is the cell count at day 5 (*t*_5_), and *N*_1_ is the cell count at day 1 (*t*_1_).

### 2.6 Algal Fatty Acids

We used gas chromatography with mass selective detection (GC–MS) to analyse the fatty acid (FA) composition of *Pseudokirchneriella*. Samples were centrifuged at 1600 g for 6 min by Heraeus Megafuge 16 (Thermo Fisher, Germany), and the algal pellet was removed, frozen at −20°C, and then lyophilized. Lyophilized cells (2–5 mg) were weighed into tin cups (Elemental microanalysis, UK), and algal lipids were extracted with chloroform‐methanol (2:1 by volume) according to Folch et al. (1957). Extracted lipids were transmethylated with 1% sulfuric acid in methanol at 90°C for 90 min (Strandberg et al. 2015, 2022). The fatty acid methyl esters were analysed with an Agilent 6890N GC equipped with Agilent 5973N MS. The column was DB‐23 (60 m × 0.25 mm × 0.25 μm). We used helium as the carrier gas with an average velocity of 26 cm/s. The injection mode was splitless, and the inlet temperature was 250°C. The initial oven temperature was 50°C, which was held for 1 min, after which the temperature was increased at 15°C/min to 150°C, followed by 0.50°C/min to 170°C, and finally 2°C/min to 230°C, which was held for 2 min. Identification and quantification of fatty acids were based on mass spectra and reference standard GLC‐538 (Nu‐Chek‐Prep). Data are presented as weight percentages (w%). Additionally, the total fatty acid content in algae is presented as micrograms per milligram dry weight (μg/mg DW).

### 2.7 Size‐Exclusion Chromatography (HPSEC) and Total Organic Carbon—Total Nitrogen (TOC–TN) Analyses

We used high‐performance size exclusion chromatography (HPSEC) with fluorescence detection to analyse the molecular size distribution and abundance of dissolved organic compounds in the media. Sample preparation protocol, parameters of the TOC–TN analysis and HPSEC separation, and the details of data processing were conducted following the methods described in the previous study (Ignatev and Tuhkanen 2019). In this study, we followed UVA254 and the following fluorescence responses: tyrosine‐like (TYR) at λex/em = 220/310 nm, tryptophan‐like (TRP) at λex/em = 270/355 nm, fulvic‐like (FUL) at λex/em = 330/425 nm, and humic‐like (HUM) at λex/em = 390/500 nm.

### 2.8 Statistical Analyses

Univariate two‐way ANOVA was used to test the differences in cell growth and total FA content of *Pseudokirchneriella*. In the ANOVA, temperature (two levels) and NP treatments (four levels) were used as independent fixed factors with type III sum of squares. In addition to the main effects, the interaction effect of temperature and NP treatments was tested. We used partial Eta‐squared as the effect size. We tested the homogeneity of variances with Levene's test and normality with the one‐sample Kolmogorov–Smirnov test, using an alpha‐level of 0.01. Both the specific growth rate and fatty acid data met the assumption of normal distribution and homogeneity of variances.

Multivariate analyses were used to evaluate the differences in algal fatty acid profiles (weight%) between temperature and NPs treatments. Before the analyses, the proportional fatty acid data was arcsine square root transformed. We used a distance‐based test for homogeneity of multivariate dispersions of the dataset (PERMDISP), and permutational multivariate analysis of variance (PERMANOVA) to test differences in algal fatty acid profiles between the treatments (Anderson et al. 2008). Additionally, we used similarity percentages (SIMPER) to identify which fatty acid contributed the most to the variation (Clarke 1993). We used non‐metric multidimensional scaling to depict the differences in fatty acid profiles between treatments (Clarke 1993). In the PERMANOVA, we used the same two fixed factors as for the ANOVA, that is, temperature with two levels (20°C and 25°C), and NPs exposure with four levels (control, NPs 1: low dose, NPs 2: medium dose, and NPs 3: high dose). We used Bray–Curtis similarity as the resemblance matrix. Total fatty acid content was used as a covariate in the analysis. Because we used total FA content as a covariate, permutations were conducted under a reduced model using type I (sequential) sum of squares. The number of permutations was 9999. We used 0.01 as the alpha level. We also calculated the effect size from the estimates of components of variation (Anderson et al. 2008). Negative estimates of components of variation were treated as zero. Multivariate analyses were conducted with Primer 6.1.15 with PERMANOVA +1.0.5 add‐on (Primer‐e), and univariate analyses were conducted with SPSS Statistics 27.0.1.0 (IBM Corporation). For HPSEC data, *p* values were calculated in Microsoft Excel using the *t*‐Ttest function (two‐tailed distribution, two‐sample equal variance).

## 3 Results

### 3.1 NP Characteristics

**FIGURE 1:**
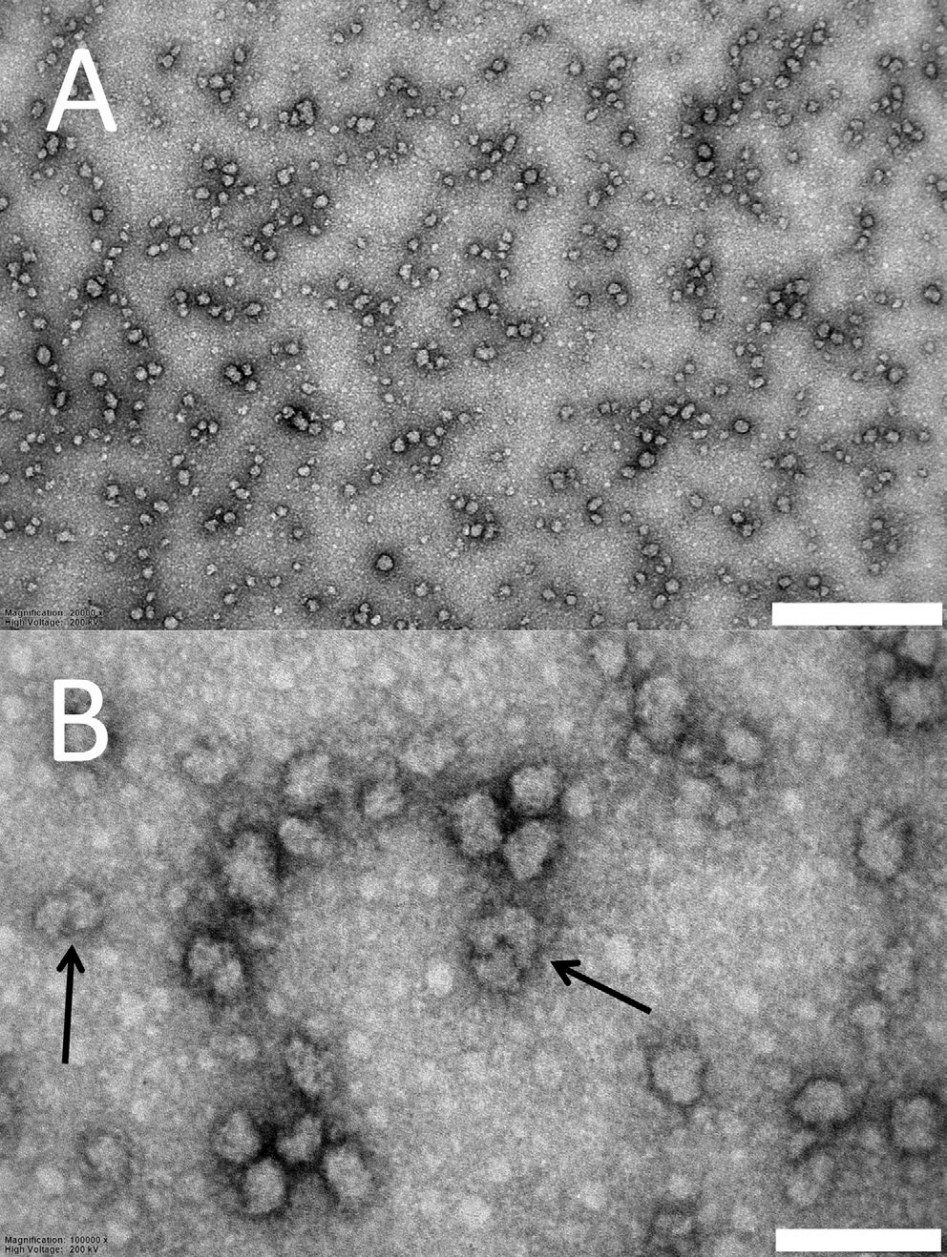
TEM micrographs of NPs of studied specimens. (A) An Overview of NPs imaged using magnification of 20 kx. Scalebar length equals 500 nm and (B) detailed view revealing the morphology of NPs imaged using 100 kx. Locations of agglomerated particles are indicated with arrows. Also, uneven background due to formvar film morphology can be seen. True NPs have dark surroundings due to negative staining. Scalebar length equals to 100 nm.

Typical TEM micrographs of negative‐stained NPs are shown in Figure 1. The particle size of diluted NP specimens was estimated using TEM image analysis. The mean particle diameter of the NPs obtained using TEM was (34 ± 7) nm. The particle diameter minimum and maximum were 17 nm and 50 nm, respectively. The effective particle size was measured using the DLS method from a 5.0 mg/mL solution and was (58 ± 32) nm. Nanoplastics in TEM specimens could be studied individually. The morphology of most NPs was round or elliptical in shape (Figure 1A). However, there were fine structures on the NP surfaces (Figure 1B), and some particles had shapes like a star or the letter L. Additionally, we found some agglomerated NPs, indicated by arrows (Figure 1B).

### 3.2 Growth

**FIGURE 2:**
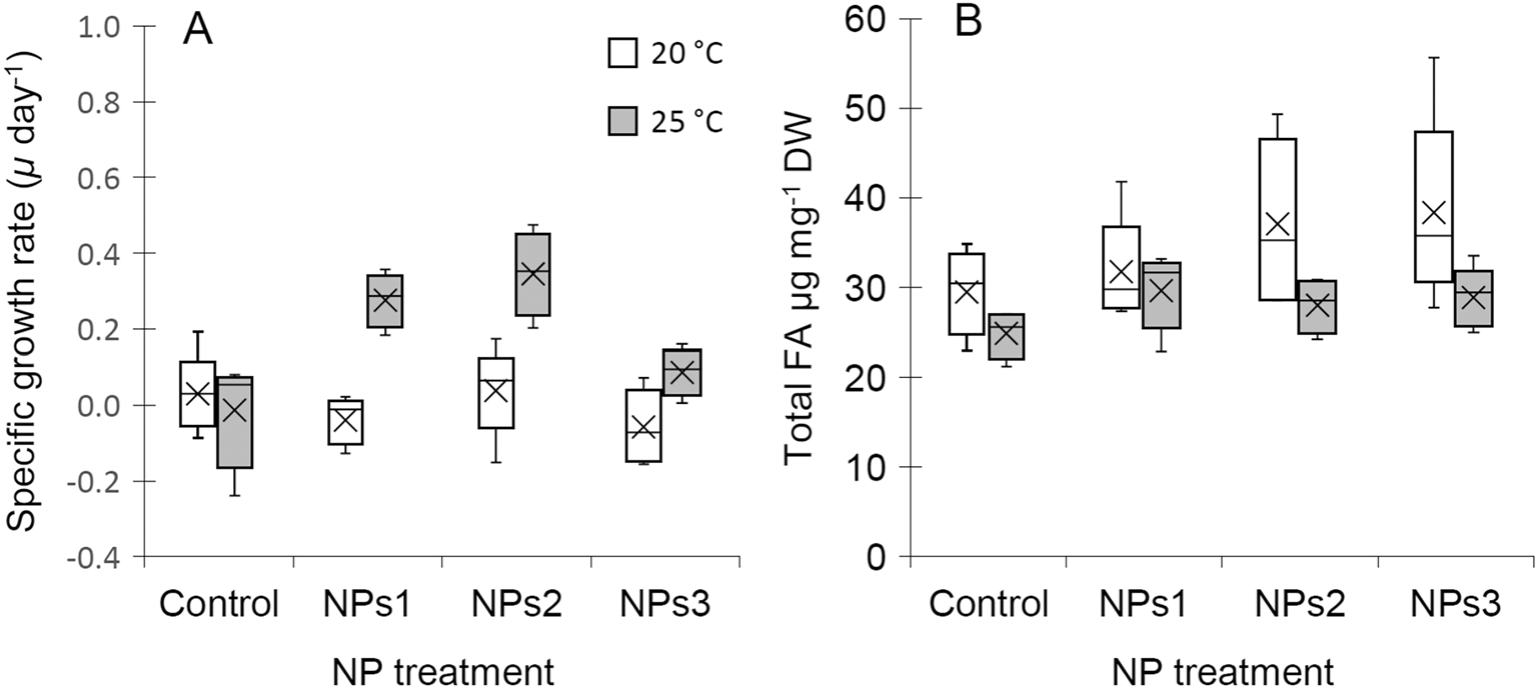
Specific growth rate (A) and total fatty acid content (B) in *Pseudokirchneriella* at 20°C and 25°C in different nanoplastics (NPs) treatment.

The mean specific growth rate of *Pseudokirchneriella* throughout the NP exposures at 20°C was zero, and at 25°C the mean specific growth rate was 0.17 (Figure 2A). A significant interaction effect was found between temperature and nanoplastic treatments, which accounted for 39.6% of the variation in the specific growth rates of *Pseudokirchneriella* (Table S1 and Figure 2A). The specific growth rates between NP treatments did not differ at 20°C, but at 25°C, algae grew faster when exposed to low and moderate NP concentrations (i.e., the NPs 1 and NPs 2) (Table S1 and Figure 2A). The specific growth rates did not differ between the control and NPs 3.

### 3.3 Fatty Acids

The total fatty acid content in *Pseudokirchneriella* ranged from 20.9 to 54.9 μg mg^−1^ DW. Temperature had a significant effect on the total fatty acid content (Table S2); the mean total fatty acid content was 33.7 μg/mg DW at 20°C, and 27.7 μg/mg DW at 25°C across all nanoplastic additions. Temperature accounted for 24.2% of the variation in total FA content in *Pseudokirchneriella* (Table S2). The nanoplastic treatments had no effect on the total FA content in *Pseudokirchneriella*, nor was there any interaction effect between temperature and NP treatments (Table S2). Although there seemed to be rather high variation in the NP treatments at 20°C (Figure 2B), the variances of total FA content in *Pseudokirchneriella* between treatments were homogeneous (Levene's test, *p* = 0.121).

**FIGURE 3:**
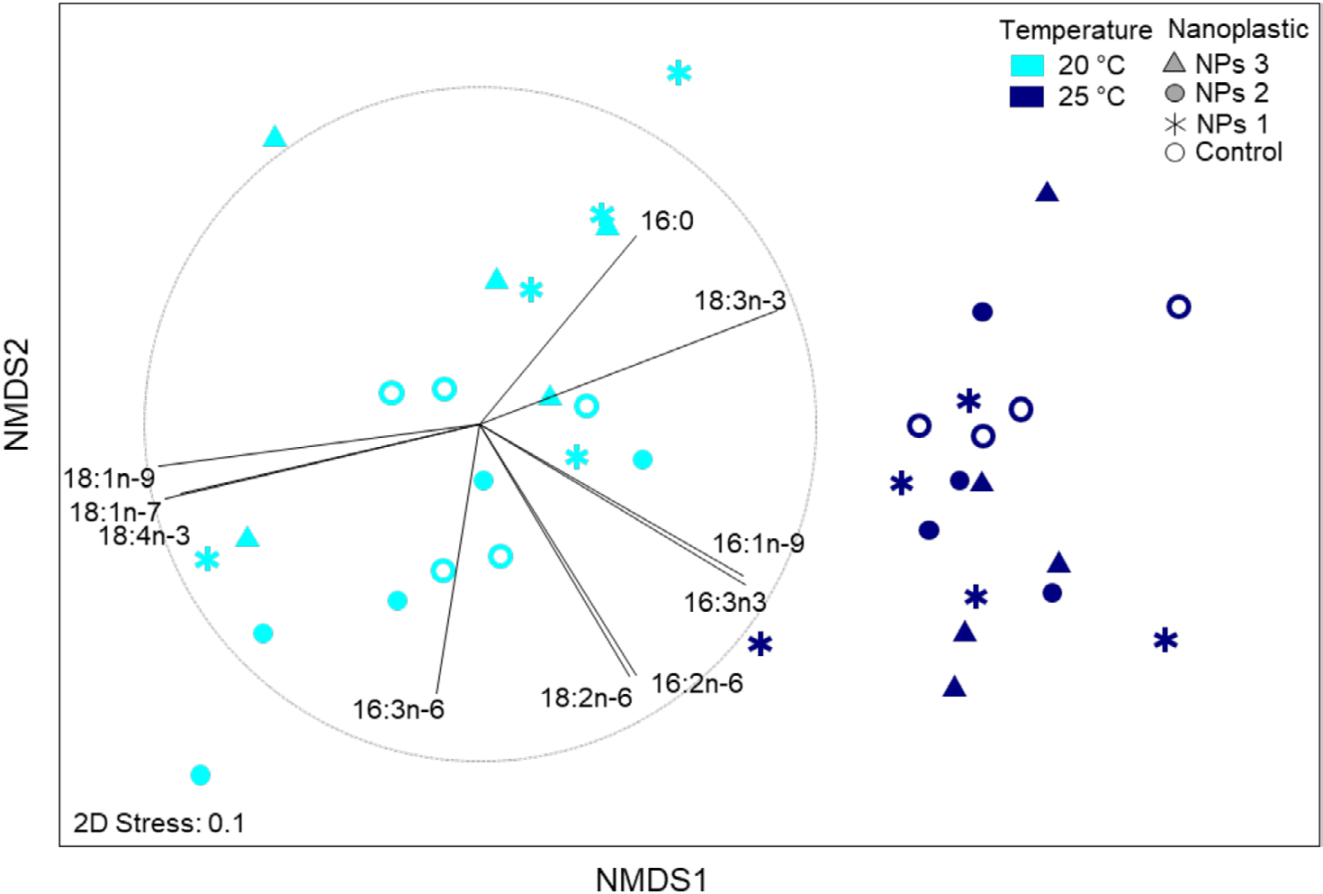
Non‐metric multidimensional scaling of fatty acid profile (w%) of *Pseudokirchneriella* in different temperature and NP treatments. Fatty acids that correlate strongly (*r* > 0.5) with either of the dimensions are presented. Vectors indicate the strength and direction of the correlation between fatty acid and dimension.

We identified 19 fatty acids in *Pseudokirchneriella*, and the most abundant fatty acids were 18:3n‐3, 16:0, 16:4n‐3, and 18:1n‐9 (Table S3). These fatty acids accounted for 80.2%–83.3% of fatty acids in *Pseudokirchneriella* across all temperature and NPs treatments. The multivariate dispersions of fatty acid w% were homogeneous between the treatments (PERMDISP *p* = 0.763). The PERMANOVA analysis of the algal fatty acid profiles showed that both experimental treatments, that is, temperature and NPs, as well as the covariate, that is, total FA content, had a significant effect on the FA w% (Table S4). The total FA content in *Pseudokirchneriella* could explain 13% of the variation in the fatty acid profiles. After the effect of total FA had been accounted for, the experimental temperature could explain 51% of the variation in the fatty acid profiles in *Pseudokirchneriella* (Table S4). The fatty acid profiles of *Pseudokirchneriella* differed between temperature treatments in all the NPs levels (ESM), as also seen in Figure 2B. The fatty acids contributing the most to the dissimilarity between temperature treatments were 18:1n‐9 (18.1%), 18:3n‐3 (9.8%), 18:1n‐7 (9.4%), 16:3n‐3 (9.3%), 16:1n‐9 (8.2%), and 18:4n‐3 (6.9%). Altogether, these six fatty acids accounted for 61.6% of the dissimilarity in the fatty acid profiles of *Pseudokirchneriella* between the temperature treatments. The acute temperature rise, from 20°C to 25°C, resulted in a decline in w% of 18:1n‐7, 18:1n‐9, and 18:4n‐3, and an increase in w% of 18:3n‐3, 16:3n‐3, and 16:1n‐9 in *Pseudokirchneriella* (Table S3 and Figure 3).

The nanoplastic treatment did not have a strong effect on *Pseudokirchneriella* fatty acid profiles. Nanoplastic additions could explain only 4% of the variation in the fatty acid profiles. Interactions between treatments and covariates were not significant, although note the *p* = 0.025, with effect size 0.07 (Table S4). Pair‐wise analysis showed that the fatty acid profiles differed only between two treatments, that is, NPs 2 and NPs 3 at 20°C (Table S5). The fatty acids that contributed the most to the dissimilarity between NPs 2 and NPs 3 at 20°C were 18:1n‐9 (13.6%), 14:1 (12.2%), 18:3n‐3 (7.4%), and 17:0 (7.3%). Fatty acids 14:1 and 17:0 are present in trace amounts in the samples (< 0.2% of all fatty acids). Additionally, 17:0 is also known to be a bacterial fatty acid, thus reflecting the differences in bacteria in the samples. Note that the mean sum of odd‐chained fatty acids, 15:0 and 17:0, was 0.1%. Thus, although the algae cultures were not axenic, the very low contribution of these bacterial fatty acids indicates a low bacterial biomass in all the samples (Strandberg et al. 2020). The magnitude of the difference in the fatty acid profiles between the NPs 2 and NPs 3 treatments at 20°C was very low, and as mentioned above, the effect size was small (Table S4); thus, the observed difference between NPs 2 and NPs 3 treatments at 20°C for *Pseudokirchneriella* is probably not physiologically or ecologically meaningful.

### 3.4 Fractionation of AOM With Size‐Exclusion Chromatography (HPSEC)

**FIGURE 4:**
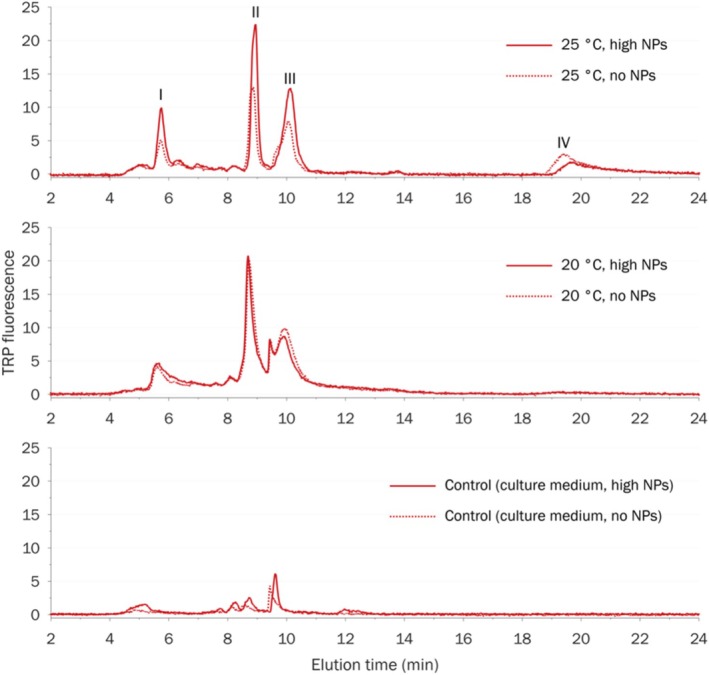
HPSEC chromatogram with TRP‐fluorescence detection (*λ*_ex/em_ = 270/355 nm).

**FIGURE 5:**
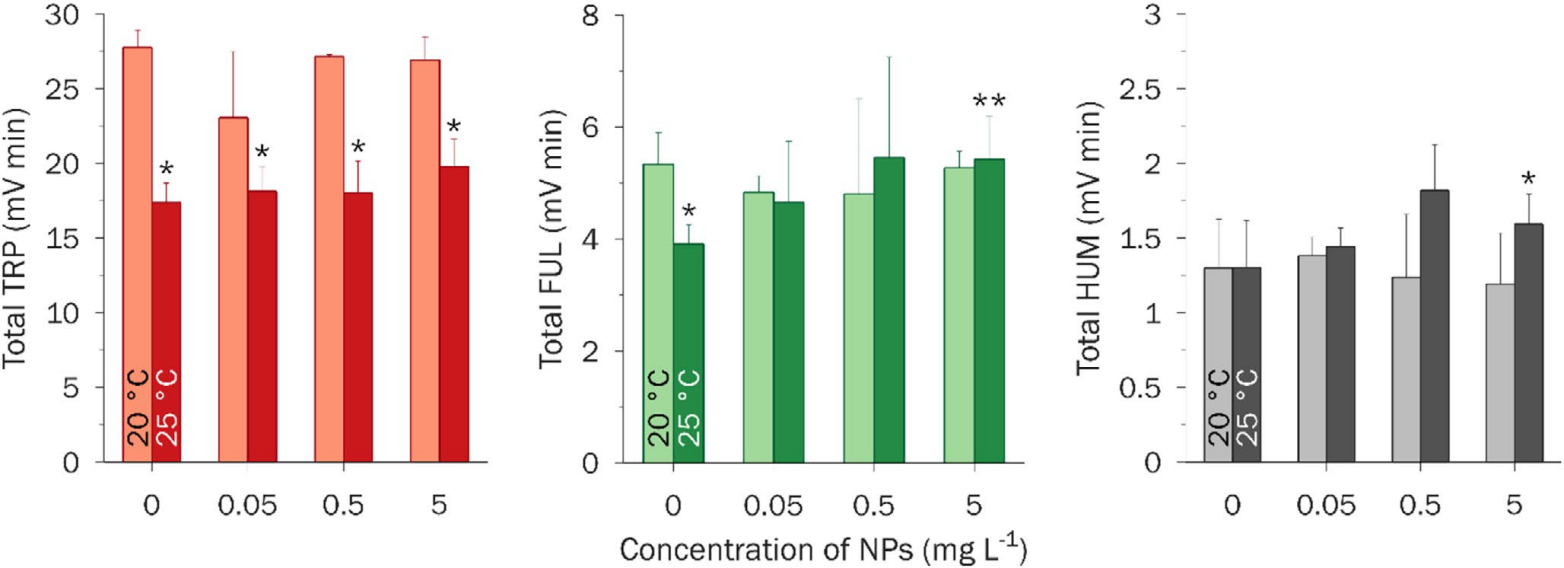
Total fluorescence intensities of whole (unfractionated) AOM samples. Mean ± SD (*n* = 5). Statistically significant difference (*p* < 0.05) is marked with * for treatment pairs with the same concentration of NPs, but different temperatures (i.e., 5 mg L^−1^ and 25° vs. 5 mg L^−1^ and 20°), and with ** for different concentrations of NPs, but the same temperature (i.e., 5 mg L^−1^ and 25° vs. 0 mg L^−1^ and 25°).

**FIGURE 6:**
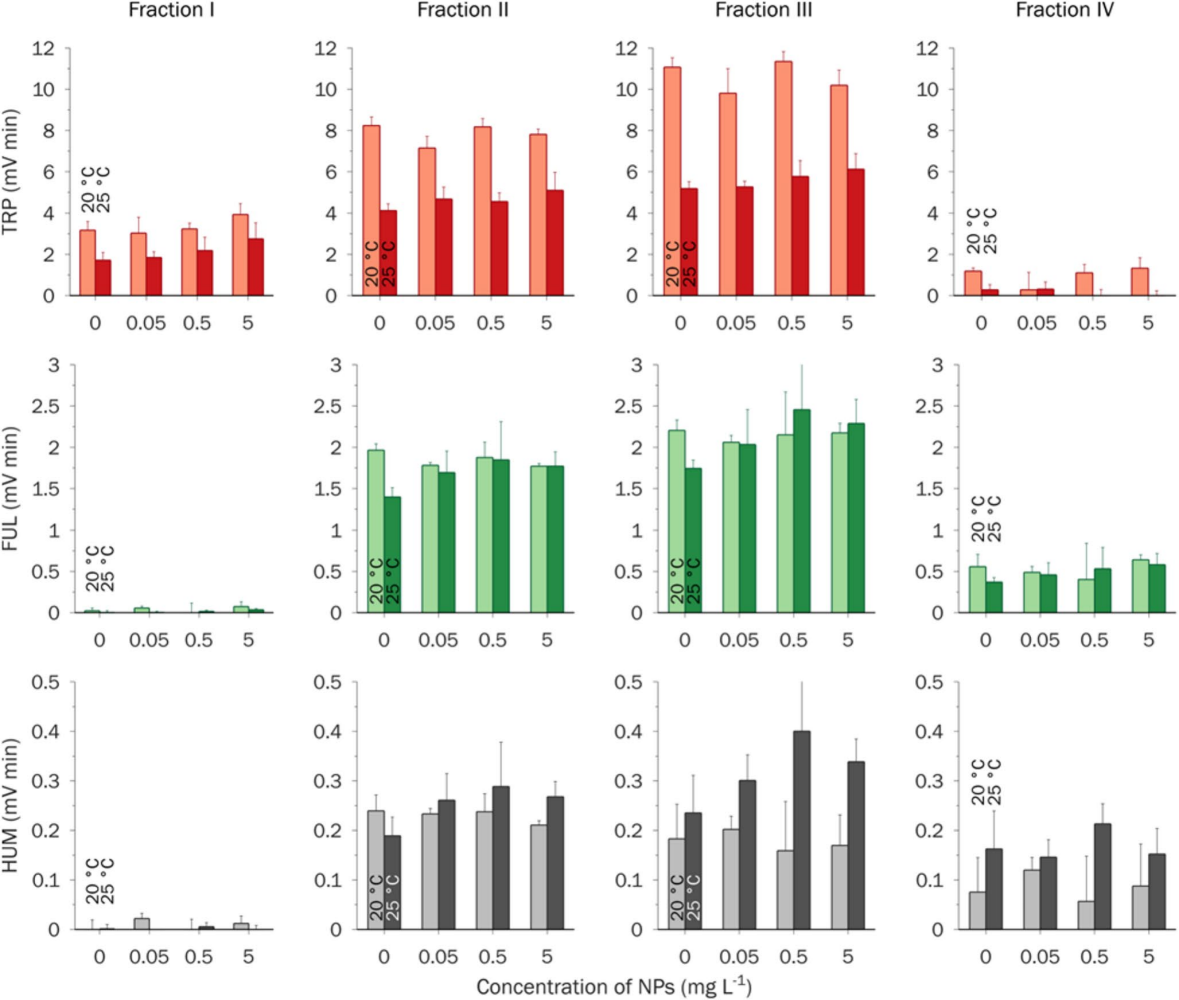
Fluorescence intensities of individual HPSEC fractions I–IV of AOM. Mean ± SD (*n* = 5).

All the AOM samples had strong TRP‐fluorescence, which significantly exceeded the fluorescence responses of control samples (Figure 4). The absolute intensities of FUL and, especially, HUM fluorescence were much lower (Figures S1 and S2). None of the AOM samples exhibited TYR fluorescence (Figure S3). The total fluorescence intensities (Table S6 and Figure 5), calculated as the sum of all chromatographic peak areas of a sample, represent the total fluorescence responses of the whole (unfractionated) sample. UVA254 signal of the algal organic matter (AOM) samples did not exceed that of control samples (culture media in the presence and absence of NPs but without algal cells) (Figure S4). Moreover, the area of HPSEC‐UVA254 fraction eluted at 9.5 min decreased during the experiment. Concentration effects of NPs were observed only at 25°C: an increase in the concentration of NPs correlated with a moderate increase in the total TRP (by less than 10%) and total FUL fluorescence (by less than 20%). To calibrate the size‐exclusion column, we used a range of narrow MW polystyrene sulphonate standards (PSS). As an additional standard, we used a reference fulvic acid 2S103F, isolated by IHSS, with a weight‐average MW of 1.4 kDa and number‐average MW of 0.9 kDa (Ateia et al. 2017) (Figure S5). Permeation volume was determined with acetone. In all HPSEC chromatograms of AOM samples, resolved peaks were deliberately combined into 4 fractions with apparent molecular weight (MW) decreasing from fraction I to fraction III. Fraction IV eluted outside the calibration range (elution times between 4 and 12 min); thus, no estimation of MW can be given for this fraction. Fraction I (elution time 5–6 min) with the highest MW > 10 kDa exhibited a moderate TRP‐fluorescence but no FUL/HUM fluorescence. This fraction is typically associated with extracellular biopolymers, mostly polysaccharides (up to 90%) and proteins (up to 10% during the exponential growth phase and up to 30% during the stationary phase) (Villacorte et al. 2015). No difference in the fraction I area was observed between the two temperatures. However, in the presence of NPs, the area of fraction I increased by 30%–50% (Figure 6). Fraction II (elution time 9 min) and III (elution time 10 min) with intermediate to low MW < 1 kDa had high TRP‐fluorescence, moderate FUL fluorescence and low HUM fluorescence. Fraction II was attributed to building blocks and low MW fulvic/humic‐like substances, while fraction III was associated with low MW acids and neutrals (weakly and uncharged organic compounds) (Villacorte et al. 2015; Huber et al. 2011). FUL and HUM fluorescence of fractions II and III was the same for both temperatures in the presence and absence of NPs (Figure 6). However, the fractional TRP‐fluorescence exhibited a two‐fold decrease under the higher temperature of 25°C. Moreover, at 25°C in the presence of a high concentration of NPs, TRP‐fluorescence of fractions II and III increased by 20%–25% compared to the control. Fraction IV eluted outside the calibration range of elution time 4–12 min, and estimation of its MW was not possible. In HPSEC separation, late elution is often caused by specific (e.g., hydrophobic) interactions between sample components and the column material or ion exclusion of cationic compounds (Le Coupannec et al. 2000). TRP‐fluorescence of this fraction in the samples exposed to the higher temperature of 25°C was 8–10 times higher than in the samples exposed to the lower temperature of 20°C, regardless of the concentration of NPs. At the same time, neither FUL nor HUM fluorescence demonstrated a significant difference.

### 3.5 The Total Organic Carbon (TOC) and Total Nitrogen (TN) Contents

According to the protocol of TOC/TN analysis, the samples were filtered through 0.45 μm filters, which retained the algal cells but allowed the NPs to pass. Thus, the reported TOC values include carbon content not only from AOM but also from the NPs. However, some NPs might have adsorbed on the algal cells during exposure, resulting in their retention on the filter. The fraction of the adsorbed NPs is unknown. Thus, we calculated the differences in TOC content at 25°C vs. 20°C for all concentrations of NPs (Figure S6). Assuming that the possible adsorption was the same at both temperatures, the observed differences in TOC content indicate slightly more intense production of AOM at 25°C (by 0.25 ± 0.28 mgC/L at the NPs concentration of 5 mg/L and by 0.17 ± 0.08 mgC/L at the NPs concentration of 0.05 mg/L). Unfortunately, the amount of AOM was too low for reliable quantification using the available TOC analyser. The averaged contents of TOC and TN of AOM produced at both temperatures in the presence and absence of NPs can be found in Tables S7 and S8.

The TN content is also consistently higher for the AOM samples produced at the higher temperature. However, a statistically significant difference (14.3 ± 2.5 mgN/L) was observed only at the concentration of NPs of 0.05 mgC/L.

## 4 Discussion

The results of this study suggest that the combined effects of the acute temperature increase and NPs exposure differed depending on the response variable, that is, growth, fatty acid composition or AOM composition. At lower temperature, NPs exposure did not affect algal growth. However, the acute temperature increase stimulated algal growth at low to intermediate NP concentrations, but not at the highest NP concentrations. In contrast, we did not observe any such interaction effect on the fatty acid data. The acute temperature increase altered the fatty acid composition, but NPs exposure had no significant effect on the fatty acids in green algae. AOM produced during algal growth at the lower temperature had higher TRP‐fluorescence (especially for low MW fractions), but similar FUL and HUM fluorescence compared to the higher temperature. According to TOC measurements, AOM production was slightly higher in the presence of NPs at the higher temperature. According to TN measurements, the consumption of N from the culture medium was higher at the lower temperature.

### 4.1 Characterisation of NPs

Characterisation using TEM and DLS revealed the size and shape of the studied NPs. However, the size estimate obtained with DLS was larger than the size measured using TEM imaging. This discrepancy can be explained by the probable agglomeration of NPs after sonication just before the DLS measurement. During TEM imaging, the uneven background due to the formvar film morphology could lead to mistakenly identifying tiny 10 nm dots as nanoparticles. However, true NPs should have a dark surrounding in TEM micrographs due to negative staining and can be easily recognised.

### 4.2 NPs And Warming Effects on Growth

Here, we examined the growth response (mass production) to warming and NPs, and their combination. Temperature and NPs alone had no impact on growth, but the combined effects were observed at NP concentrations of 0.05 and 0.5 mg/L, indicating that the combined effects of NPs and high temperatures may have an impact on algae growth. To date, there are no reports on the combined effects of warming and NPs on the growth of green algae, particularly *Pseudokirchneriella*. In fact, studies on the effects of nano/microscale PMMA polymer on algae are scarce and contradictory, most likely due to differences in experimental parameters, such as particle size, algal species, and/or temperature (Venâncio et al. 2019; Li, Li, Li, Zhao, Geng, Sun, et al. 2020; Li, Li, Li, Zhao, et al. 2020; Sun et al. 2023). A previous study found that 5 days of exposure to 1 μm MPs at three environmentally relevant concentrations (0.1, 1, and 10 mg/L) increased growth at 25°C in the marine pennate diatom, *Phaeodactylum tricornutum* (Sun et al. 2023). Interestingly, higher temperature alone had the opposite effect, reducing algal growth. However, MPs have been shown to affect organisms in the presence of other stressors, even if they do not have any effects alone (Li, Li, Li, Zhao, Geng, Sun, et al. 2020; Li, Li, Li, Zhao, et al. 2020). A 96 h exposure to 40 nm PMMA‐NPs significantly decreased the growth rate among four marine algae species at the optimal temperature of 20°C (Venâncio et al. 2019). Specifically, the growth of *Tetraselmis chuii* and *Nannochloropsis gaditana* was reduced at concentrations higher than 150 mg/L, while *Thalassiosira weissflogii* and *Isochrysis galbana* showed decreased growth at concentrations equal to and higher than 18.8 mg/L and 213.6 mg/L, respectively. On the other hand, PMMA MPs (1 μm) slightly increased the growth of microalgae *Gymnodinium aeruginosum* after a 48 h exposure to high concentration (75 mg/L) (Huang et al. 2021). Interestingly, this growth‐promoting effect was not observed with smaller sizes of particles; possibly the larger particles provide a substrate for algae to grow on.

However, more attention has been given to the impacts of polystyrene (PS) plastic particles on algae. 72 h exposure to polystyrene 50 nm NPs has also reduced growth by 57% in green algae *Dunaliella tertiolecta* , but only at high concentrations (250 mg/L) (Sjollema et al. 2016).

Feng et al. (2020) observed a reduction in the growth of cyanobacteria *Microcystis aeruginosa* by 23.57% and 46.10% after 72 h of exposure to NPs at low (3.40 μg/mL) and high (6.80 μg/mL) concentrations, respectively. These findings suggest that PS may be more toxic than PMMA.

McKeel et al. (2024) have indicated that nanoparticle‐induced aggregation of algal cells can alter growth, cell division, and photosynthetic activity. Species‐specific sensitivity and morphological characteristics should be considered, as these factors may interact with the physical and chemical properties of NPs and potentially influence their toxicity (Venâncio et al. 2019). The observed growth increase in our study may also be attributed to the synergistic effects of warming and NPs. Nanoplastics, acting as a stressor, could have triggered a response in the algae, leading to increased growth. Furthermore, it can also be explained by the biphasic dose–response known as hormesis, where aquatic organisms exhibit contrasting responses at low and high concentrations (Agathokleous et al. 2021). These responses can be induced by various factors such as the type of plastic polymers, nanoparticle sizes, and shapes.

### 4.3 NPs And Warming on Fatty Acid Content and Profiles

The acute temperature stress, but not NPs, had a significant negative effect on the total fatty acid content of *Pseudokirchneriella*. Moreover, we did not observe any interactive effects of NPs and temperature. The results also indicated that the total fatty acid content responded to the acute temperature increase in less than 5 days. Most previous experiments have used temperature‐acclimatised algal cultures, and our knowledge of the response time of fatty acid content to temperature stress is very limited (Nalley et al. 2018). The temperature dependence of total fatty acid content and fatty acid composition has been documented in most algal taxa, including green algae (Singh and Singh 2015; Nalley et al. 2018). However, thermal stress may have positive or negative effects on total fatty acid content (Singh and Singh 2015; Nalley et al. 2018; Feijão et al. 2018). Generally, the growth rate increased with temperature until the species‐specific growth optimum temperature was reached, and the highest total FAs content was observed at temperatures close to the growth optimum (Nalley et al. 2018).

The fatty acid composition of *Pseudokirchneriella* was typical for green algae; the most abundant fatty acids were 18:3n‐3, 16:0, 16:4n‐3, and 18:1n‐9 (Taipale et al. 2013). Contrary to our hypothesis, we did not observe any systematic increase in saturated fatty acids at higher temperatures. Instead, the strongest temperature responses, both negative and positive, were observed for monounsaturated and polyunsaturated fatty acids. Specifically, the w% of 18:1n‐7, 18:1n‐9, and 18:4n‐3 decreased with the acute temperature stress, while the w% of 18:3n‐3, 16:3n‐3, and 16:1n‐9 increased. Previously, increased temperatures have been linked to a decreased proportion of polyunsaturated fatty acids in algae, as a response to maintain membrane lipid semifluidity at higher temperatures (Sushchik et al. 2003; Sharma et al. 2012). For instance, a temperature increase from 20°C to 28°C decreased the proportion of n‐3 PUFAs and increased the proportion of saturated FAs (16:0 and 18:0) in the green algae *Scenedesmus obliquus* (Fuschino et al. 2011). This is known as homeoviscous adaptation (Hazel 1995). However, exceptions to this general pattern have been documented for some algal taxa (Sharma et al. 2012; Nalley et al. 2018). For example, a temperature increase from 21°C to 29°C had no effect on the total FA content in the green algae *Derbesia tenuissima* (Gosch et al. 2015). The discrepancies between taxa may be related to the overall abundance of saturated fatty acids in the algae and the necessity for homeoviscous adaptation. Additionally, membrane lipids have many physiological functions, such as in the electron transfer chain in thylakoid membranes, which may constrain possible fatty acid modifications (Wada and Murata 1990). The temperature‐mediated decrease of n‐3 polyunsaturated fatty acids, as well as the n‐3/n‐6 ratio, may be linked with the proper function of the photosystem II complex and the electron transfer system (Wada and Murata 1990; Strandberg et al. 2021). The nanoplastics had no strong effects on the FA profile, with differences observed only between the concentrations of 0.5 and 5 mg/L. While previous studies on *Pseudokirchneriella* are limited, changes in the FA profile after exposure to NPs at high concentrations (0.05 and 5 μg/mL) have been reported in diatoms (Gonzalez‐Fernandez et al., González‐Fernández et al. 2020). Specifically, they observed increased proportions of 18:1n‐7, 16:2n‐4, and 16:3n‐4, suggesting some metabolic responses in polyunsaturated fatty acid (PUFA) pathways. Currently, no research has examined the combined impact of warming and NPs on algal fatty acid content. However, a study on the combined effects of warming and MPs found that exposure to MPs (1 μm) combined with warming (25°C compared with 21°C) increased the biosynthesis of unsaturated fatty acids and glycerolipid metabolism in the marine diatom *P. tricornutum* (Sun et al. 2023). The increased FA biosynthesis could potentially be a response to low nutrient availability due to the increased growth rate at higher temperatures. Guschina et al. (2020) found that exposure to < 70 μm MPs altered lipid synthesis in the green algae *Chlorella sorokiniana*, resulting in a reduction in essential FAs. Changes in lipid biosynthesis and fatty acid composition in algae could also impact food quality, growth, and stress resistance in primary consumers. Research on the effects of PMMA on algae FAs is limited, making it challenging to draw direct comparisons with our findings. Nonetheless, the toxicity of plastic particles can vary, depending on size and dose effects (Zhao et al. 2020; Yang et al. 2020). Notably, higher concentrations of smaller PS and PMMA may lead to more pronounced effects on algae (Huang et al. 2021).

Our results indicated that NPs and the acute temperature increase promoted the growth of *Pseudokirchneriella* without increasing the total fatty acid content, although the acute temperature stress modified fatty acid profiles. This suggests that temperature plays a more critical role in altering fatty acid composition than exposure to NPs. In this experiment, we simulated the acute temperature stress, which may occur in small ponds and lakes during heatwaves. Increased biomass due to algal responses to combined stress factors may suggest a potential increase in resource quantity for zooplankton. The nutritional quality, in terms of n‐3 PUFA, did not respond negatively to the temperature and NP stressors, at least in the short term. Long‐term studies, however, have documented decreased n‐3 PUFA levels (Fuschino et al. 2011; Guschina et al. 2020). This could have consequences for the higher trophic levels, including fish and other aquatic organisms, affecting their growth and reproduction (Gladyshev et al. 2009). It should also be noted that our study focused on a single algal species, and responses to combined stressors may differ across taxa or within algal communities. Future studies at the multi‐species or community level are needed to better assess the ecological relevance of our findings.

### 4.4 NPs And Warming Effects on Fractionation of AOM

Our findings demonstrated that the TRP‐fluorescence of AOM samples at 20°C was 20%–30% lower than that of AOM samples at 20°C. This statistically significant difference was observed at all NP concentrations. Our findings showed that NP concentrations higher than 0.05 mg/L at 25°C caused a slight increase in total TRP (< 10%) and total FUL fluorescence (< 20%). These changes are likely attributed to NPs, which stimulated an additional stress response, further intensifying the metabolic suppression induced by high temperature. It is well documented that numerous algal species exhibit a rapid response, releasing significant amounts of extracellular proteins and carbohydrates within hours, as a self‐defence mechanism when exposed to various stressors such as organic compounds, heavy metals, and nanoparticles (Maršálek and Rojíčková 1996; Quigg et al. 2013). Khan et al. (2022) found that TRP‐fluorescence significantly correlated with the number of disintegrated algae cells during ultrasonication. TRP, FUL, and HUM are known for their fluorescence properties. Tryptophan is an essential amino acid that plays a crucial role in protein synthesis and various metabolic pathways. FUL and HUM fluorescence typically refer to compounds resembling humic and fulvic substances. TRP fluorophores are present in fulvic and humic isolates. Here, a reference fulvic acid, isolated from peat by IHSS, exhibited a noticeable TRP‐fluorescence (Figure S5). At the same time, pure proteins did not display FUL and HUM fluorescence. The absence of TYR fluorescence suggests that neither free amino acid tyrosine nor heavily degraded proteinaceous material was formed because tyrosine units in proteins and peptides do not emit fluorescence in the presence of tryptophan units (Yamashita and Tanoue 2003; Lobus et al. 2022). Assuming that the fluorescent AOM formed in different treatments had similar chemical compositions and quantum yields, we can conclude that the algal cells produced 20%–30% more protein‐like compounds (according to the total TRP‐fluorescence) at 20°C than at 25°C in all NP concentrations. One possible explanation is a reduced metabolism at a higher temperature. Another possible cause could be an initial thermal shock as the algal cells were transferred from a 20°C stock solution to the culture medium heated to 25°C without a gradual temperature adjustment. However, no significant differences were observed for the total FUL and total HUM fluorescence between the temperatures, suggesting that the formation of fulvic‐ and humic‐like AOM was not affected at the higher temperature. The decrease in the UVA254 of the fraction eluting at 9.5 min could be attributed to the consumption of the culture medium's organic ingredients (such as vitamins B1, B12, etc.) by growing algal cells.

We observed that the TRP‐fluorescence of fractions II and III increased at high temperature. This substantial increase in low MW organics might be an indication of cell lysis and the release of intracellular materials to the AOM pool (Villacorte et al. 2015). Based on HPSEC‐fluorescence analysis, it can be suggested that the higher temperature significantly inhibited the release of protein‐like AOM with MW < 1 kDa (fractions II and III), but did not change the fluorescence intensity of high MW protein‐like biopolymer fraction I. The presence of NPs at higher temperature partly offset this temperature effect and increased the TRP‐fluorescence of HPSEC fractions II and III.

## 5 Conclusions

This study highlights the complex interplay between the acute temperature increases and NP exposure on green algae growth and fatty acid content. The findings indicate that while the acute temperature increase can enhance algal cell growth at low to intermediate NP concentrations, higher NP concentration reverses this effect. Temperature increase altered the fatty acid composition, but NP exposure did not affect the fatty acids. Additionally, the production of AOM varied with temperature, showing increased TRP‐fluorescence at the lower temperature, while NP presence at the higher temperature slightly increased the AOM production as indicated by TOC measurements. These results emphasise the necessity for further research to elucidate the underlying mechanisms, particularly considering environmental changes and their implications for algal physiology and ecosystem dynamics.

## Author Contributions

**Sareh Yaripour:** conceptualization, methodology, formal analysis, investigation, software, data curation, validation, visualization, writing – original draft, writing – review and editing. **Sadikshya Ghimire:** investigation. **Alexey Ignatev:** methodology, formal analysis, investigation, software, data curation, validation, visualization, writing – review and editing. **Raine Kortet:** funding acquisition, resources, writing – review and editing. **Rebecca Burkl:** investigation. **Jari T. T. Leskinen:** methodology, data curation, formal analysis, resources, writing – review and editing. **Jussi V. K. Kukkonen:** funding acquisition, writing – review and editing. **Jarkko Akkanen:** resources, writing – review and editing. **Jukka Kekäläinen:** resources, writing – review and editing. **Mohammad Salar Sohrabi:** investigation. **Ursula Strandberg:** methodology, formal analysis, software, validation, resources, supervision, funding acquisition, writing – review and editing.

## Funding

This work was supported by Saastamoisen säätiö, Jenny ja Antti Wihurin Rahasto, OLVI‐Säätiö, and Academy of Finland (338261, 346541).

## Conflicts of Interest

The authors declare no conflicts of interest.

## Supporting information


**Data S1:** Supporting Information.

## Data Availability

The data that supports the findings of this study areis available in the Supporting Information of this article.
