# Supplementary material for: Combined Impact of Nanoplastics and Temperature on Green Algae: Implications for Growth, Lipid Content and Organic Exudates
Source: Environ Microbiol Rep. 2025 Dec 2;17(6):e70246. doi: 10.1111/1758-2229.70246 (PMC12672135; doi:10.1111/1758-2229.70246)
Supplement: Supplementary file 1 — Data S1: Supporting Information. [file EMI4-17-e70246-s001.rtf]

Supplementary information


Combined impact of nanoplastics and temperature on green algae: implications for growth, lipid content and organic exudates

Sareh Yaripour1*, Sadikshya Ghimire1$, Alexey Ignatev2$, Raine Kortet1, Rebecca Burkl1, Jari T.T. Leskinen3, Jussi V.K. Kukkonen1, Jarkko Akkanen1, Jukka Kekäläinen1, Mohammad Salar Sohrabi1, Ursula Strandberg1

1Department of Environmental and Biological Sciences, University of Eastern Finland, P.O. Box 111, FI-80101, Joensuu, Finland
2 Department of Biological and Environmental Science, University of Jyväskylä, P.O. Box 35, FI-40014, Jyväskylä, Finland
3 Department of Technical Physics, SIB Labs, University of Eastern Finland, P.O. Box 1627, FI-70210, Kuopio, Finland
$ Contributed equally

*Corresponding author, E-mail: sareh.yaripour@uef.fi; phone: +358 413139407


Table S1. Two-way ANOVA summary table for the specific growth rate in Pseudokirchneriella across different temperature and nanoplastics (NPs) treatments. Significant factors are bolded. Partial Eta-Squared was used as Effect size.
Source of variation	df	F	P	Effect size	
Temp	1	31.252	0.000	0.510	
NPs	3	7.231	0.001	0.420	
Temp × NPs	3	6.543	0.002	0.396	
Within group	30				
Total	37				


Table S2. Two-way ANOVA summary table for total FA content in Pseudokirchneriella across different temperature and nanoplastics (NPs) treatments. The significant factor is bold. Partial Eta-Squared was used as Effect size. 
Source of variation	df	F	P	Effect size	
Temp	1	9.567	0.004	0.242	
NPs	3	1.875	0.155	0.158	
Temp × NPs	3	0.771	0.519	0.072	
Within group	30				
Total	37				


Temperature	NPs	N	14:0	14:1	15:0	16:0	16:1n-9	16:1n-7	16:1n-5	
			Mean	SD	Mean	SD	Mean	SD	Mean	SD	Mean	SD	Mean	SD	Mean	SD	
20 °C	Control	5	0.25	0.02	0.03	0.01	0.08	0.02	17.53	0.46	0.59	0.02	4.33	0.08	0.13	0.02	
	NPs 1	5	0.26,	0.02	0.01	0.00	0.08	0.01	17.61	0.67	0.52	0.06	4.26	0.24	0.12	0.02	
	NPs 2	5	0.26	0.01	0.01	0.01	0.08	0.01	16.77	0.72	0.62	0.03	4.31	0.21	0.14	0.00	
	NPs 3	5	0.28	0.01	0.16	0.29	0.08	0.01	17.14	0.48	0.54	0.04	4.44	0.14	0.13	0.02	
25 °C	Control	4	0.29	0.05	0.03	0.01	0.08	0.01	17.56	0.40	0.81	0.03	4.37	0.07	0.14	0.01	
	NPs 1	5	0.25	0.02	0.02	0.00	0.08	0.01	17.39	0.27	0.83	0.05	4.52	0.08	0.25	0.20	
	NPs 2	4	0.25	0.02	0.01	0.01	0.06	0.01	17.61	0.28	0.83	0.02	4.40	0.24	0.13	0.01	
	NPs 3	5	0.27	0.02	0.01	0.01	0.08	0.01	17.61	0.43	0.88	0.04	4.45	0.25	0.15	0.02	
		N	16:2n-6	16:3n-6	17:0	16:3n-3	16:4n-3	18:0	18:1n-9	
			Mean	SD	Mean	SD	Mean	SD	Mean	SD	Mean	SD	Mean	SD	Mean	SD	
20 °C	Control	5	0.30	0.03	0.21	0.02	0.03	0.02	1.79	0.09	14.84	0.32	0.42	0.04	13.14	0.55	
	NPs 1	5	0.27	0.03	0.20	0.02	0.03	0.01	1.62	0.11	14.98	0.42	0.44	0.09	12.72	1.08	
	NPs 2	5	0.32	0.01	0.23	0.02	0.04	0.01	1.82	0.10	14.51	0.42	0.38	0.03	13.74	1.22	
	NPs 3	5	0.28	0.02	0.20	0.02	0.01	0.00	1.61	0.09	14.87	0.38	0.37	0.03	12.94	0.72	
25 °C	Contr	4	0.31	0.02	0.19	0.02	0.03	0.01	2.12	0.07	14.98	0.49	0.52	0.11	10.14	0.56	
	NPs 1	5	0.36	0.03	0.24	0.04	0.03	0.01	2.17	0.09	14.86	0.33	0.44	0.04	10.94	0.41	
	NPs 2	4	0.36	0.03	0.20	0.02	0.03	0.01	2.31	0.12	14.58	0.45	0.44	0.09	11.04	0.51	
	NPs 3	5	0.37	0.06	0.22	0.03	0.02	0.01	2.28	0.14	14.84	0.34	0.43	0.08	10.65	0.28	
		N	18:1n-7	18:2n-6	18:3n-6	18:3n-3	18:4n-3	n-6 PUFA	n-3 PUFA	
			Mean	SD	Mean	SD	Mean	SD	Mean	SD	Mean	SD	Mean	SD	Mean	SD	
20 °C	Control	5	1.19	0.06	2.16	0.09	0.11	0.04	36.15	0.45	6.71	0.16	2.79	0.15	59.49	0.56	
	NPs 1	5	1.08	0.16	1.98	0.15	0.08	0.03	36.94	0.74	6.81	0.34	2.53	0.21	60.35	0.66	
	NPs 2	5	1.26	0.13	2.30	0.11	0.11	0.01	35.97	0.51	7.12	0.31	2.97	0.12	59.42	0.64	
	NPs 3	5	1.12	0.13	2.04	0.12	0.08	0.02	36.92	0.62	6.79	0.38	2.60	0.16	60.20	0.77	
25 °C	Control	4	0.82	0.08	2.20	0.07	0.09	0.02	39.13	0.41	6.18	0.15	2.80	0.07	62.41	0.78	
	NPs 1	5	0.83	0.15	2.49	0.11	0.08	0.02	38.08	0.83	6.15	0.31	3.17	0.18	61.26	0.77	
	NPs 2	4	0.74	0.08	2.55	0.03	0.09	0.02	38.25	0.03	6.12	0.19	3.19	0.06	61.26	0.50	
	NPs 3	5	0.76	0.06	2.50	0.23	0.08	0.02	38.21	0.92	6.20	0.20	3.17	0.33	61.53	0.61	
Table S3. The mean and standard deviation of saturated (14:0, 15:0, 16:0, 17:0, 18:0), monounsaturated (14:1, 16:1n-9, 16:1n-7, 16:1n-5, 18:1n-9, 18:1n-7) and polyunsaturated (16:2n-6, 16:3n-6, 16:3n-3, 16:4n-3, 18:2n-6, 18:3n-6, 18:3n-3, 18:4n-3) fatty acids and the sum of n-6 and n-3 PUFA in Pseudokirchneriella at two experimental temperatures (20 °C and 25 °C) with different nanoplastics (NPs) additions (Control, NPs 1, NPs 2, NPs 3). Fatty acids data represent weight percentages (w%) of total fatty acids.


Table S4. PERMANOVA summary for fatty acid profile (w%) in Pseudokirchneriella across temperature and nanoplastics (NPs) treatments. Total fatty acid content (total FA) was used as a covariate. Significant factors are bolded.
Source of variation	df	Pseudo-F	Unique perms	P	Effect size	
total FA	1	21.358	9949	0.000	0.13	
Temp	1	34.074	9938	0.000	0.51	
NPs	3	2.5892	9930	0.003	0.04	
total FA × Temp	1	1.5955	9933	0.159	0.02	
total FA × NPs	3	0.98762	9928	0.472	0.00	
Temp × NPs	3	1.9934	9940	0.025	0.07	
total FA × Temp ×NPs	3	0.88001	9936	0.569	0.00	
Residual	22					
Total	37					


Table S5. Pair-wise comparisons of fatty acid profiles in Pseudokirchneriella exposed to four levels of NPs (control, NPs 1, NPs 2, NPs 3) at two experimental temperatures (20 ºC and 25 ºC)
Temperature	NP exposures	t	P(perm)	Unique perms	
20 ℃	NPs 3, NPs 2	2.427	0.000	9943	
	NPs 3, NPs 1	1.181	0.249	9941	
	NPs 3, control	1.501	0.043	9937	
	NPs 2, NPs 1	1.763	0.056	9930	
	NPs 2, control	0.728	0.717	9930	
	NPs 1, control	1.762	0.028	9920	
25 ℃	NPs 3, NPs 2	0.712	0.667	9821	
 	NPs 3, NPs 1	0.842	0.582	9949	
	NPs 3, control	1.307	0.198	9816	
	NPs 2, NPs 1	0.834	0.659	9809	
	NPs 2, control	1.840	0.039	8845	
	NPs 1, control	1.456	0.097	9838	


Table S6. P-values (two-tailed distribution, two-sample equal variance) were calculated to determine statistically significant differences between total and fractional TRP fluorescence of AOM samples. Treatments at different temperatures were compared with corresponding controls and with each other (i.e., different temperatures but the same concentration of NPs).
			Fraction					
Temperature	NP concentration	Total	I	II	II	IV	Compared to:	
	High	0.09	0.01	0.05	0.08	0.06	control-25	
		0.00	0.04	0.00	0.00	0.00	high-25	
	Medium	0.94	0.48	0.77	0.80	0.19	control-25	
25 °C		0.00	0.01	0.00	0.00	0.00	medium-20	
	Low	0.18	0.16	0.10	0.23	0.42	control-25	
		0.06	0.02	0.00	0.00	0.81	medium-20	
	Control	0.00	0.00	0.00	0.00	0.00	control-20	
	High	0.86	0.04	0.86	0.69	0.56	control-20	
20 °C	Medium	0.66	0.88	0.60	0.14	0.52	control-20	
	Low	0.06	0.59	0.06	0.14	0.03	control-20	


Table S7. The TOC (mgC/L) content of AOM produced at 25 °C and 20 °C and different concentrations of NPs (mean ± SD, n = 5).
	Concentration of NPs (mg/L)	
	0	0.05	0.5	5	
20 °C	1.42 ± 0.05	1.33 ± 0.03	1.47 ± 0.13	2.23 ± 0.12	
25 °C	1.30 ± 0.15	1.50 ± 0.10	1.50 ± 0.20	2.49 ± 0.21	


Table S8. The TN (mgN/L) content of AOM produced at 25 °C and 20 °C and different concentrations of NPs (mean ± SD, n = 5).
	Concentration of NPs (mg/L)	
	0	0.05	0.5	5	
20 °C	76.8 ± 3.4	67.8 ± 2.3	75.7 ± 4.0	74.8 ± 9.2	
25 °C	78.6 ± 5.0	82.1 ± 1.1	79.7 ± 6.7	81.1 ± 3.1	


Figures


Figure S1. HPSEC chromatogram with TYR fluorescence detection (ëex/em = 220/310 nm).


Figure S2. HPSEC chromatogram with FUL fluorescence detection (ëex/em = 330/425 nm).


Figure S3. HPSEC chromatogram with HUM fluorescence detection (ëex/em = 390/500 nm).


Figure S4. HPSEC chromatogram with UVA254 detection


 Figure S5. Comparison of normalised HPSEC chromatograms of AOM and standard fulvic acid (2S103F).


Figure S6. Changes in the contents of TOC (left) and TN (right) at 25 °C vs. 20 °C at different concentrations of NPs (mean ± SD, n = 5).


Raw Data

Table R1. Tryptophan-like fluorescence (mV*min).
		Tryptophan-like fluorescence			
			Total		Fraction			
Temperature	NP concentration	Replicate		I	II	III	IV	
25 °C	High	1	22.0719	3.1258	6.3358	7.2351	-0.2385053	
		2	18.0048	1.9213	4.5678	5.6696	0.106482	
		3	20.394	3.6403	5.099	5.9484	-0.166256	
		4	18.7677	2.3171	4.4139	5.6542	0.274465	
		5	21.7646	3.6517	5.9959	6.2153	-0.35834	
	Medium	1	20.5454	3.018	4.6599	6.5905	0.321633	
		2	15.7562	1.9563	4.1138	5.2441	-0.314115	
		3	16.8488	1.478	4.3502	4.9929	0.168429	
		4	18.9633	2.2748	5.1029	6.2611	-0.229857	
		5	2.8688	0.4548	0.5075	0.6466	0.183389	
	Low	1	18.2492	1.69	4.3769	5.5121	0.671927	
		2	16.9768	1.7068	4.1564	4.925	0.492491	
		3	16.8757	1.7276	4.7124	5.1712	-0.126746	
		4	20.4893	2.2631	5.4716	5.4701	0.1212672	
		5	22.6248	2.5694	5.5877	6.3729	0.917798	
	Control	1	17.59	1.7966	4.1823	4.9391	0.080652	
		2	3.7099	0.4073	0.6209	1.1878	0.161609	
		3	18.6543	2.2073	4.5076	5.6092	0.078697	
		4	17.7287	1.5037	4.066	5.3095	0.607476	
		5	15.5803	1.3515	3.7226	4.8949	0.357406	
20 °C	High	1	27.561	3.895	8.1259	10.2764	1.4681	
		2	26.77	4.3251	7.735	9.9196	1.1342	
		3	24.852	4.3075	7.5117	9.4155	0.7573	
		4	28.543	3.1916	7.8901	11.1638	1.9565	
		5	31.545	4.0083	9.6786	12.4702	1.4667	
	Medium	1	26.992	3.381	7.6732	10.7773	1.5076	
		2	27.217	2.8228	8.0645	11.1681	1.3552	
		3	27.287	3.3587	8.3703	11.5723	0.9401	
		4	27.188	3.3735	8.6094	11.8737	0.6227	
		5	28.272	3.4741	8.5584	11.6333	1.1225	
	Low	1	25.679	3.3628	7.5475	10.6957	0.7354	
		2	27.25	3.8848	7.6023	10.6487	1.0097	
		3	22.029	2.7576	7.0868	9.7515	0.2819	
		4	17.322	2.1078	6.3837	8.1465	-0.9054	
		5	25.683	3.1609	8.0085	10.6853	0.5824	
	Control	1	27.178	3.0226	8.4059	11.0216	1.0515	
		2	27.87	3.6073	8.1676	11.1239	1.1158	
		3	29.363	3.4018	8.6946	11.6247	1.1608	
		4	26.741	2.6473	7.7058	10.5152	1.4106	
		5	26.991	3.5687	7.5639	10.1947	1.3939	

Table R2. Fulvic-like fluorescence (mV*min).
		Fulvic-like fluorescence			
			Total		Fraction			
Temperature	NP concentration	Replicate		I	II	III	IV	
25 °C	High	1	6.0682	2.19E-02	1.9379	2.461578	0.722748	
		2	4.3242	2.50E-02	1.5426	1.881429	0.397278	
		3	5.7569	5.21E-02	1.8655	2.528152	0.588343	
		4	5.5377	4.20E-02	1.7417	2.287433	0.618354	
		5	4.7385	1.20E-02	1.8461	2.152318	0.387612	
	Medium	1	8.116	-3.23E-03	2.5381	3.54779	0.912224	
		2	4.3539	2.95E-02	1.5447	2.01426	0.371077	
		3	4.4378	8.75E-03	1.6645	2.157064	0.371795	
		4	4.902	2.70E-02	1.6451	2.106126	0.27281	
		5	0.1761	2.45E-03	0.1577	0.030712	0.020613	
	Low	1	6.1713	4.75E-04	2.0549	2.631589	0.622231	
		2	3.6267	-1.31E-02	1.4459	1.645552	0.276837	
		3	4.166	2.18E-02	1.5878	1.865766	0.426569	
		4	4.634	3.64E-03	1.6862	1.995774	0.508888	
		5	7.5359	5.42E-02	2.2246	3.043143	0.899641	
	Control	1	3.5766	6.78E-03	1.2347	1.639603	0.344957	
		2	0.3163	1.04E-02	0.1663	0.145532	0.068511	
		3	4.3438	1.65E-02	1.464	1.841502	0.45082	
		4	3.7136	1.75E-02	1.4124	1.679622	0.331244	
		5	4.0029	-3.11E-02	1.4823	1.819106	0.355257	
20 °C	High	1	5.404	0.026126	1.7988	2.281	0.6473	
		2	5.5571	0.150925	1.784	2.253	0.7222	
		3	4.8781	0.030632	1.7238	2.028	0.5781	
		4	5.247	0.089929	1.7783	2.1385	0.6143	
		5	5.9975	0.103747	2.0807	2.5456	0.6687	
	Medium	1	2.2993	-0.283117	1.62	1.4007	-0.2446	
		2	5.3928	0.052746	1.8755	2.2118	0.605	
		3	6.0212	0.026724	1.9602	2.4572	0.7128	
		4	5.5069	0.028373	2.0524	2.5366	0.5375	
		5	6.4341	0.07458	2.0228	2.5349	0.8115	
	Low	1	4.838	0.030569	1.7824	2.0903	0.5046	
		2	5.0137	0.086043	1.7911	2.1137	0.5297	
		3	5.0576	0.047574	1.8201	2.1042	0.5369	
		4	4.4244	0.058637	1.7358	1.9377	0.3836	
		5	5.049	-0.010444	1.85	2.1105	0.5054	
	Control	1	4.7225	-0.017366	1.9742	2.1063	0.3603	
		2	5.3886	0.03587	1.959	2.2506	0.5944	
		3	6.0814	0.01102	2.0575	2.3635	0.7198	
		4	5.1306	0.061726	1.8694	2.1014	0.5547	
		5	5.22	0.088158	1.856	2.0925	0.6001	


Table R3. Humic-like fluorescence (mV*min).
		Humic-like fluorescence			
			Total		Fraction			
Temperature	NP concentration	Replicate		I	II	III	IV	
25 °C	High	1	1.59E+00	-7.77E-03	0.27441	0.337647	0.123402	
		2	1.36E+00	3.58E-03	0.223124	0.27863	0.120402	
		3	1.58E+00	-9.73E-03	0.282012	0.348595	0.13563	
		4	1.85E+00	9.60E-03	0.29225	0.389617	0.229523	
		5	1.85E+00	7.36E-03	0.300681	0.416107	0.179206	
	Medium	1	2.22E+00	-3.41E-03	0.421674	0.5666331	0.227846	
		2	1.58E+00	6.54E-03	0.228558	0.313304	0.194579	
		3	1.58E+00	3.15E-03	0.259642	0.361081	0.168695	
		4	1.90E+00	1.60E-02	0.244687	0.360752	0.26206	
		5	2.15E-01	4.37E-03	0.030647	-0.0338561	0.066884	
	Low	1	1.45E+00	-2.14E-03	0.336776	0.367045	0.105053	
		2	1.36E+00	-6.26E-04	0.212857	0.246563	0.161922	
		3	1.34E+00	-8.56E-04	0.235307	0.276821	0.131082	
		4	1.61E+00	-1.38E-03	0.259213	0.312944	0.185758	
		5	1.88E+00	-1.29E-02	0.36894	0.465635	0.173081	
	Control	1	1.29E+00	2.20E-03	0.160515	0.222364	0.175994	
		2	1.77E-03	-5.79E-03	0.016554	-0.074472	0.016835	
		3	1.14E+00	-3.30E-03	0.176739	0.198275	0.105052	
		4	1.04E+00	-7.02E-03	0.17445	0.175559	0.103285	
		5	1.75E+00	1.32E-02	0.244709	0.345443	0.266012	
20 °C	High	1	1.2479	-5.45E-03	0.2079	0.168071	0.091467	
		2	1.4046	2.20E-02	0.2218	0.216526	0.138909	
		3	1.4262	2.69E-02	0.2123	0.211149	0.153538	
		4	0.6962	5.06E-03	0.2009	0.083298	-0.033116	
		5	1.1928	-1.40E-02	0.2521	0.170896	0.024265	
	Medium	1	0.6689	-5.14E-02	0.1966	0.024521	-0.0758222	
		2	1.1684	2.11E-04	0.2214	0.1415129	0.071513	
		3	1.5503	3.44E-03	0.253	0.2211	0.126263	
		4	1.5664	1.35E-02	0.28	0.245177	0.104915	
		5	1.177	-1.31E-02	0.2489	0.164508	0.037286	
	Low	1	1.3995	1.03E-02	0.2316	0.193141	0.126602	
		2	1.3334	1.85E-02	0.2247	0.195342	0.108643	
		3	1.5446	3.53E-02	0.2493	0.240412	0.151805	
		4	1.2553	2.43E-02	0.2281	0.179735	0.092185	
		5	1.3588	-9.19E-03	0.226	0.177025	0.097402	
	Control	1	1.7132	2.09E-02	0.2644	0.251623	0.171364	
		2	1.1683	-1.29E-02	0.2397	0.167069	0.040499	
		3	1.3789	3.14E-03	0.26	0.221122	0.078328	
		4	0.9427	-4.29E-02	0.1939	0.092451	0.011489	
		5	1.3113	-5.95E-04	0.2197	0.172228	0.109309	


Cell Count (10^5)	
Table R4. Cell count in Pseudokirchneriella across different temperature and nanoplastics (NPs) treatments.

Temperature	Flask No	NP concentration	Day 1	Day 2	Day 3	Day 4	Day 5	
25 ℃	1	High	1.76	5.9	3.2	4.4	1.8	
	2	High	0.9	2.2	3.1	3.3	1.5	
	3	High	1.6	5.4	1.6	2.3	1.9	
	4	High	1.3	2	3.7	2.1	1.9	
	5	High
	1.16	1.8	3.5	2.7	2.2	
	6	Medium	0.7	3.6	2.7	3	3.1	
	7	Medium	1.16	1.1	1.3	1.2	2.6	
	8	Medium	0.9	2.8	2	3.6	3.4	
	9	Medium	0.66	3.5	4	2.3	4.4	
	10	Medium	0.3	0.6	0	0	0	
	11	Low	1.53	4	1.4	3	5.6	
	12	Low	0.86	1.5	4.5	2.4	2.7	
	13	Low	0.6	2.2	1.96	1.9	2.5	
	14	Low	1.2	4	2.6	3	2.5	
	15	Low	1.1	1.5	2.7	2.7	2.7	
	16	Control	1.13	2.3	1	5	1.4	
	17	Control	0	1.9	0	0	0	
	18	Control	1.6	1.5	1.1	3.2	2.2	
	19	Control	1.7	2	2.1	4.8	2.1	
	20	Control	5.2	2.2	1.	1.7	2	
20 ℃	1	High	3.7	3.3	2	3.5	2.1	
	2	High	3.1	2.1	1.9	2	3.2	
	3	High	2.8	1.5	2.6	1.1	1.5	
	4	High	2	3.1	2.1	2.4	1.5	
	5	High	2.4	1.8	4	2.6	3.2	
	6	Medium	3.3	1.3	2.7	1.9	1.8	
	7	Medium	1.6	2.6	2.2	1.9	1.8	
	8	Medium	1.7	1.4	2.7	2.1	2.2	
	9	Medium	1.3	2.1	2.9	2.4	2.6	
	10	Medium	1.7	2.7	1.9	2.1	2.3	
	11	Low	2.3	2.3	2.3	1.7	2.2	
	12	Low	2.1	2.2	1.8	1.9	2.1	
	13	Low	2.2	4.1	2.4	1.5	1.6	
	14	Low	2.3	3.1	2.1	1.6	2.5	
	15	Low	2.5	2.3	2.9	1.4	1.5	
	16	Control	2.7	1.8	2	3.1	1.9	
	17	Control	1.3	2.1	2.7	2	2.8	
	18	Control	2.1	2.1	1.4	1.7	1.9	
	19	Control	2.5	1	1.6	2.5	2.9	
	20	Control	2.4	2.4	2.4	2.4	2.7	
